# Biopsy-Driven Synovial Pathophenotyping in RA: A New Approach to Personalized Treatment

**DOI:** 10.3390/jpm15120622

**Published:** 2025-12-11

**Authors:** Sheyda Ketabchi, Edda Russo, Maurizio Benucci, Maria Infantino, Mariangela Manfredi, Emanuele Antonio Maria Cassarà, Francesca Li Gobbi, Alessandro Mannoni, Riccardo Terenzi

**Affiliations:** 1 Department of Oncology, Section of Pathological Anatomy, San Giovanni Di Dio Hospital, Azienda USL Toscana Centro, 50143 Florence, Italy; sheyda.ketabchi@uslcentro.toscana.it; 2 Clinical Pathology Laboratory Unit, S. Giuseppe Hospital, Azienda USL Toscana Centro, 50053 Empoli, Italy; edda.russo@uslcentro.toscana.it; 3 Rheumatology Unit, S. Giovanni Di Dio Hospital, Azienda USL Toscana Centro, 50143 Florence, Italy; maurizio.benucci@uslcentro.toscana.it (M.B.); emanueleantonio.cassara@uslcentro.toscana.it (E.A.M.C.); francesca.ligobbi@uslcentro.toscana.it (F.L.G.); 4 Immunology and Allergology Laboratory Unit, S. Giovanni Di Dio Hospital, Azienda USL Toscana Centro, 50143 Florence, Italy; maria2.infantino@uslcentro.toscana.it (M.I.); mariangela.manfredi@uslcentro.toscana.it (M.M.); 5 Rheumatology Unit, P. Palagi Hospital, Azienda USL Toscana Centro, 50122 Florence, Italy; alessandro.mannoni@uslcentro.toscana.it

**Keywords:** rheumatoid arthritis, ultrasound-guided biopsy, synovial pathotypes, difficult to treat rheumatoid arthritis (D2T RA), Krenn score, precision medicine, molecular profiling

## Abstract

The diagnosis and treatment of rheumatoid arthritis (RA) have been constantly evolving for decades, pointing towards early diagnostic and therapeutic interventions. Synovial biopsy has emerged as a pivotal tool in precision medicine, transitioning from a research procedure to a clinically feasible approach. Modern ultrasound-guided techniques allow safe, reproducible access to inflamed joints, enabling direct analysis of the synovial tissue, which reveals biological heterogeneity undetectable in peripheral blood. Histological scoring, including the Krenn synovitis score, discriminates inflammatory from non-inflammatory pathology, supporting targeted escalation of immunosuppressive therapy. Molecular and histological profiling has defined distinct synovial pathotypes—lympho-myeloid, diffuse-myeloid, and fibroid/pauci-immune—with reproducible associations to therapeutic responsiveness. Moreover, biopsy-driven trials, such as R4RA and STRAP, demonstrate that pathotype-guided strategies can predict outcomes: diffuse-myeloid synovitis responds to IL-6 receptor blockade, lympho-myeloid synovitis to B cell depletion, and fibroid synovitis exhibits multidrug resistance. In difficult-to-treat RA, synovial biopsy differentiates inflammatory from non-inflammatory drivers of persistent symptoms, providing a rational basis for therapy selection. Ongoing biomarker-driven initiatives, including PRECISion and 3TR Precis-The-RA, aim to embed biopsy findings into clinical decision-making. In this review, it is underscored that the integration of histology, molecular profiling, and clinical context positions synovial biopsy as a patient-centered precision approach, guiding individualized therapy and bridging RA stratification with clinical practice.

## 1. Introduction

Rheumatoid arthritis (RA) is a chronic autoimmune disease marked by persistent joint inflammation leading to pain, stiffness, and progressive joint damage. It results from a deregulated immune response involving multiple inflammatory cells and mediators. Without appropriate treatment, the disease can cause significant disability and systemic manifestations. Early and targeted interventions are essential to limit progression and improve long-term outcomes. In this scenario, synovial biopsy has evolved from a procedure purely performed in research settings to a feasible clinical tool that brings RA management closer to personalized medicine. Ultrasound-guided techniques have standardized and become accessible for any kind of synovial tissue, enabling safe and reproducible sampling even in multicenter settings. Direct analysis of the diseased target tissue provides unparalleled insights into the biological heterogeneity of RA, revealing pathophysiological mechanisms that remain undetectable through peripheral blood-based assessments. Histological scoring, such as the Krenn system, allows for highly specific distinction between various components of an inflammatory synovial process, ensuring that escalation of immunosuppressive therapy in rheumatic joint diseases is targeted only to patients with true active synovitis.

Beyond this diagnostic role, molecular and histological analyses have revealed that RA is not a single disease but comprises distinct synovial pathotypes. The classification into lympho-myeloid, diffuse-myeloid, and pauci-immune/fibroblastic subtypes—based on semi-quantitative assessment of lymphoid B cells, plasma cells, lymphoid T cells, and macrophages—has proven to be reproducible and clinically meaningful. Importantly, biopsy-driven trials such as R4RA and STRAP have shown that these pathotypes predict therapeutic outcomes: diffuse-myeloid synovitis responds preferentially to IL-6R blockade, lympho-myeloid synovitis retains responsiveness to B cell depletion, while fibroid/pauci-immune synovitis is consistently linked to multidrug resistance. Bulk and single-cell RNA sequencing further refine this stratification, identifying gene expression programs that serve as predictive biomarkers of response or resistance.

These advances highlight synovial biopsy as more than a diagnostic tool—it becomes a precision procedure that integrates histology, molecular profiling, and clinical context to guide treatment choices in individual patients. In difficult-to-treat RA, biopsy helps to distinguish primary inflammation from non-inflammatory drivers of symptoms (such as degenerative joint diseases, post-traumatic arthritis, etc.), and provides a rational basis for personalized therapy selection. Ongoing biomarker-driven trials, such as PRECISion and 3TR Precis-The-RA, are now testing whether synovial profiling can be embedded into treatment algorithms. If validated, synovial biopsy will represent a cornerstone of precision medicine in RA, transforming therapeutic decision-making from trial-and-error to patient-tailored strategies.

In the present review, we provide a comprehensive synthesis of the principal strengths and emerging roles of modern synovial biopsy techniques within the framework of personalized medicine. We first discuss recent methodological advances that have refined procedural accuracy, standardization, and patient safety, thereby consolidating the validity of synovial biopsy as a reliable clinical and research tool. The integration of histological and molecular profiling into current diagnostic algorithms is then examined, emphasizing the contribution of synovial tissue analysis to the precise stratification of patients with inflammatory joint diseases. Furthermore, we highlight accumulating evidence that synovial biomarkers hold predictive value for therapeutic response, supporting a more individualized approach to treatment selection. Finally, we outline the future perspectives of synovial biopsy, considering its potential as a key platform for biomarker discovery, drug development, and the advancement of personalized strategies in rheumatology.

## 2. Synovial Biopsy Techniques

Synovial biopsy has progressed from early blind needle approaches, such as the Parker–Pearson technique in the 1960s [1], which demonstrated feasibility but suffered from variability and limited precision [2,3], to modern methods that combine safety, reproducibility, and high tissue quality. Arthroscopy and mini-arthroscopy subsequently allowed direct visualization of inflamed areas, producing reliable specimens suitable for histology and molecular assays [4,5], although the invasiveness and need for specialized facilities limited their routine use. In recent years, ultrasound-guided (US-guided) synovial biopsy has emerged as the preferred minimally invasive technique. By combining real-time imaging with semi-automatic core needles, this approach ensures accurate sampling of inflamed tissue with excellent yield across both large and small joints [6,7,8]. Its ability to access small joints, including the wrist and metacarpophalangeal joints, has broadened applicability in early disease [9]. Moreover, US is an agile and bed-side technique, personalizing the target joint to assess every RA patient. A major advance was the methodological validation by Humby and colleagues, who demonstrated that US-guided biopsy could achieve results comparable to arthroscopy while being far less invasive [10]. Prospective series confirmed that the procedure is safe, well tolerated, and associated only with mild, transient effects, with no significant adverse events reported [6,7,8,10]. These data established US-guided biopsy as feasible in outpatient settings, where it can be performed under local anesthesia in less than 30 min, enabling serial sampling for longitudinal studies. Building on these technical developments, EULAR–OMERACT initiatives have defined consensus recommendations on tissue handling and reporting standards [11,12], and structured training curricula, including a standardized EULAR model validated for large- and small-joint biopsies, now support reproducible implementation across centers [13]. Together, these advances have transformed synovial biopsy into a safe, standardized, and clinically feasible tool, providing the methodological foundation for biopsy-driven research in RA.

## 3. From Synovial Immune Cell Infiltration to Synovial Pathotype Definition

Histological studies have long highlighted heterogeneity in the inflammatory infiltrate of RA synovium. A key development came from Manzo et al., who showed that chemokines such as CXCL13 and CCL21 orchestrate ectopic lymphoid neogenesis, explaining the biological basis of B cell-rich synovial inflammation [14]. In parallel, Krenn and colleagues developed a three-component histopathological synovitis score, based on lining hyperplasia, stromal activation, and inflammatory infiltrate (each 0–3 points; total 0–9 points). In a large validation study, the score discriminated low-grade changes, typical of degenerative disease and post-traumatic arthritis, from high-grade synovitis seen in primarily inflammatory arthritis, with excellent inter-observer reproducibility [15]. Further work linked score components to molecular markers (MMP1, CD3, CD138, CD14), supporting its biological validity [16]. Independent studies confirmed that the Krenn score is reproducible and applicable in daily pathology practice [17].

Synovial biopsies from RA patients can also be categorized by their B cell content, with tissues described as B cell-rich when CD20^+^ B cells and CD138^+^ plasma cells are abundant, or B cell-poor when these populations are sparse [14,18]. While this distinction is biologically meaningful, it does not capture the variability of the myeloid lineage infiltrate, and CD68^+^ macrophages in particular, which represent a major component of synovial inflammation. This limitation prompted the move toward a more refined classification that integrates both lymphoid and myeloid markers, forming the basis for the definition of synovial pathotypes [15,16,17,18].

The next advance was the systematic definition of pathotypes in the Pathobiology of Early Arthritis Cohort (PEAC). Humby et al. combined histology, immunohistochemistry, and transcriptomics in DMARD-naïve RA patients [18]. At least six biopsy fragments per patient were evaluated to reduce sampling error. Sections were stained for CD20, CD3, CD138, and CD68, and each marker was semi-quantitatively scored on a 0–4 scale (0 = absent, 1 = mild, 2 = moderate, 3 = marked, 4 = very marked). Classification followed these thresholds:

- Lympho-myeloid: CD20 ≥ 2 and/or CD138 ≥ 2, with abundant CD68-sublining macrophages (≥2).
- Diffuse-myeloid: CD20 ≤ 1, CD138 ≤ 1, and CD68-sublining ≥ 2, with variable CD3^+^ T cells.
- Fibroid (pauci-immune): CD20, CD3, and CD138 < 1, with CD68 limited to the lining.

**Figure 1:**
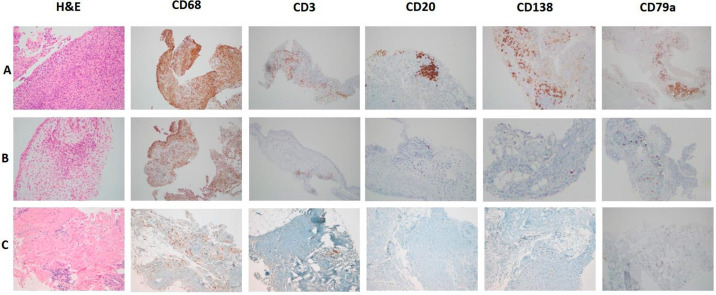
A case series of different RA synovial pathotypes from our center is shown in Figure 1 ((**A**): lympho-myeloid pattern, (**B**): diffuse-myeloid pattern, (**C**): fibroid pattern).

A case series of different RA synovial pathotypes from our center is shown in Figure 1. These categories were reproducible across centers and associated with clinical outcomes, and are now consistently used in translational studies and trials as the standard tissue-based stratification framework in RA [18].

## 4. Synovial Pathotypes as Determinants of Treatment Response: Evidence from Clinical Trials

The Krenn synovitis score is a validated histological tool that integrates enlargement of the lining cell layer, the cellular density of the synovial stroma, and leukocytic infiltrates. It allows for reliable discrimination between rheumatic (synovitis score of ≥5: high grade synovitis) and non-rheumatic joint diseases with a specificity exceeding 95% and with a sensitivity of 61.7%. However, while it provides excellent diagnostic accuracy, the score offers no information regarding differential response to therapy.

To address this limitation, biopsy-driven clinical trials have explored whether histological pathotypes and molecular signatures can guide treatment selection.

Earlier mechanistic biopsy studies provided proof that synovial tissue is a sensitive readout of drug action. Trials with anti-TNF agents demonstrated that a rapid decline in CD68^+^ sublining macrophages, evident within 48 h, is a reproducible pharmacodynamic marker associated with clinical improvement [19]. Abatacept studies showed transcriptomic “deactivation” of inflammation without depletion of infiltrating cells, indicating pathway-specific effects [20]. Likewise, short-term trials of tofacitinib and tocilizumab demonstrated suppression of JAK/STAT and IL-6-driven transcriptional networks, correlating with subsequent clinical response [21,22]. Chemokine genes such as CXCL9, CXCL10, and CXCL13, involved in lymphocyte trafficking and ectopic lymphoid structure formation, were consistently down-regulated in responders, underscoring their role as markers of lymphocyte homing and treatment sensitivity. These early observations provided proof-of-mechanism and established the synovial tissue’s key role for drug action, paving the way for more recent biopsy-driven stratified trials.

The R4RA randomized controlled trial represented a turning point. Patients with inadequate response to TNF inhibitors were randomized to rituximab or tocilizumab after baseline biopsy. Importantly, the simple dichotomic histologic classification into “B cell-rich” versus “B cell-poor” did not predict response to either rituximab or tocilizumab. Instead, stratification by more granular pathotype showed that diffuse-myeloid synovia responded better to tocilizumab than to rituximab, whereas lympho-myeloid tissues—bearing B cell-linked programs—displayed similar benefit from both agents [23]. In the extension study, Rivellese et al. integrated histology with bulk RNA sequencing and demonstrated that IL-6-responsive genes (e.g., SOCS3, STAT3 targets, CCL2, IL1B) were enriched in diffuse-myeloid tissues responsive to tocilizumab, while immunoglobulin and CXCL13 transcripts predicted benefit from rituximab in lympho-myeloid synovia. Fibroid tissues were characterized by stromal/fibroblast programs (HOX family, ECM remodeling genes) and associated with multidrug resistance [24].

The following STRAP trial substantially confirmed R4RA results in AR biologic-naive patients: the STRAP trial, comprising two parallel, open-label, biopsy-driven, phase 3 studies in biologic-naive patients aimed to evaluate whether synovial B cell signatures could guide therapy selection [25]. In the clinical phase, 226 patients were randomized to rituximab, tocilizumab, or etanercept, stratified by B cell-poor versus -rich synovitis. The primary endpoint—16-week ACR20 response in the B cell-poor population—was not met, as no significant differences were observed between rituximab and etanercept/tocilizumab combined (60% vs. 59%, OR 1.02, *p* = 0.97), highlighting the limited predictive value of the dichotomous B cell classification in biologic-naive patients. Nevertheless, the extension study performed deep molecular profiling of baseline synovial biopsies using RNA sequencing, revealing gene signatures and single-cell clusters predictive of response or resistance to rituximab, tocilizumab, or etanercept. Machine learning models derived from these data accurately predicted individual drug responses, demonstrating that multidimensional molecular and cellular analyses of the synovium can uncover treatment-relevant heterogeneity beyond conventional histology.

The STRAP trial extension study confirmed that transcriptomic profiling of synovial tissue is a central strategy for uncovering the molecular heterogeneity of RA [26]. RNA-sequencing demonstrated that baseline gene-expression programs segregate robustly into the canonical myeloid, lymphoid, and fibroid synovial pathotypes. The myeloid pathotype was enriched for inflammatory mediators such as IL1B, IL6, and CXCL5, and the lymphoid pathotype showed high expression of B cell-associated genes including MS4A1, CD79A, and CXCL13, while the fibroid pathotype was dominated by stromal and extracellular-matrix programs such as COL1A1 and THBS2. Beyond bulk RNA-seq, single-cell transcriptomics (scRNA-seq) has further refined our understanding of cellular and functional organization within the RA synovium. Particularly, STRAP demonstrated that B cell and Tfh-like clusters enriched for CXCL9, CXCL10, and CXCL13 predicted rituximab response; myeloid clusters with IL-6-responsive signatures aligned with tocilizumab benefit; and fibroblast-derived clusters with ECM and developmental genes (HOX, COL1A1, COL3A1) correlated with treatment resistance [26]. These studies firmly establish that integration of histology with bulk and single-cell transcriptomics enables prediction of therapeutic sensitivity and resistance in RA, providing a rational framework for precision medicine. Earlier seminal scRNA-seq works [27] first identified distinct fibroblast lineages, including inflammatory THY1^+^ sublining fibroblasts and PRG4^+^ lining fibroblasts, revealing their differential expansion in inflamed tissue. Subsequent atlases [28] have mapped pathogenic macrophage subsets (e.g., HBEGF^+^ macrophages), stromal activation states, and T cell phenotypes, further demonstrating that these cellular configurations align with the molecular pathotypes defined by transcriptomics. Interestingly, speculative data on abatacept show transcriptomic profiling of synovial biopsies similar to those of other DMARDs. Particularly, tocilizumab exhibited the highest concordance of modulated genes (r = 0.71), suggesting that the drug could be an option for treating RA patients expressing the lympho-myeloid pathotype [29].

Overall, bulk and single-cell transcriptomics constitute the most mature omic framework in RA synovial research. Together, they delineate how gene-expression programs organize inflammatory circuits, map onto canonical synovial pathotypes, and support precision-medicine strategies aimed at matching patients to targeted therapies based on synovial tissue biology.

The pivotal genes related to different RA pathotypes have been summarized in Table 1.

## 5. Difficult-to-Treat Rheumatoid Arthritis: Definition, Challenges, and Role of Synovial Biopsy in Today’s Personalized Therapy

The concept of difficult-to-treat rheumatoid arthritis (D2T RA) has been formalized by EULAR as the presence of at least the following:

(i) Treatment failure with ≥2 biologic or targeted synthetic DMARDs (with different mechanisms of action) after inadequate response to conventional DMARDs;
(ii) Presence of active/progressive disease despite these therapies;
(iii) Problematic management defined by the treating rheumatologist and/or the patient [30]. This definition underscores the heterogeneity of D2T RA, which is not a synonym of refractory inflammation but rather a complex and multifactorial condition.

Epidemiological data show that up to 40% of RA patients fail to achieve adequate response to first-line conventional synthetic DMARDs, most commonly methotrexate [31]. Among those who escalate to biologics or JAK inhibitors, a significant proportion continue to have insufficient response or lose efficacy over time, contributing to the pool of D2T patients [32]. However, only a subset of these individuals have true ongoing synovial inflammation. Others meet the D2T definition due to different factors such as residual pain and central sensitization, structural damage, comorbidities (e.g., fibromyalgia, cardiovascular disease, obesity), or intolerance and adverse events to multiple agents. Distinguishing inflammatory from non-inflammatory drivers of persistent disease is therefore essential.

In this setting, synovial biopsy offers unique opportunities. First, the Krenn synovitis score allows reliable discrimination of active synovitis versus non-inflammatory synovial proliferation, ensuring that escalation of immunosuppression is targeted only to patients with histologically proven inflammation.

Second, for patients with confirmed active synovitis, histological and molecular pathotype classification (lympho-myeloid, diffuse-myeloid, fibroid) provides additional guidance on expected therapeutic responsiveness.

As demonstrated in the R4RA and STRAP trials, diffuse-myeloid synovia predict better outcomes with IL-6R blockade, lympho-myeloid synovia retain responsiveness to B cell-directed therapies, while fibroid synovia enriched in stromal programs are associated with resistance across mechanisms.

**Figure 2:**
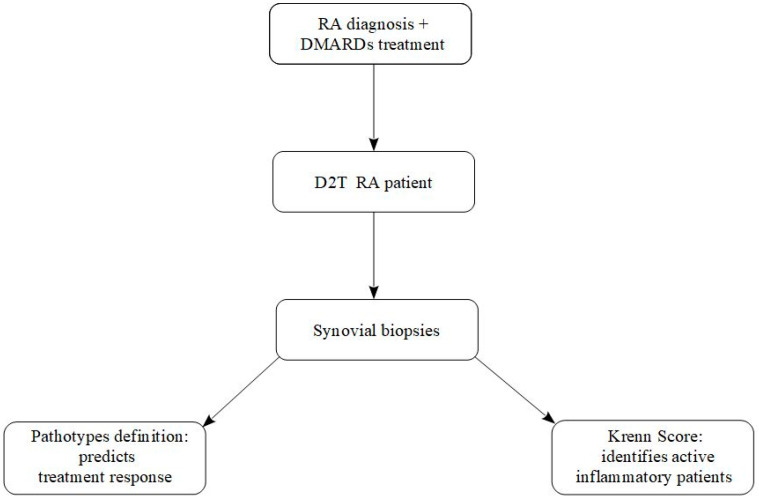
A schematic algorithm of RA synovial biopsy’s positioning in today’s practice.

Thus, in D2T RA, synovial biopsy can serve two complementary functions: (i) diagnostic—confirming whether persistent symptoms reflect primarily inflammatory process; and (ii) predictive—guiding rational selection of second- or third-line therapies based on synovial pathotype. The concept is schematized in Figure 2.

Incorporating biopsy into the evaluation of D2T patients may refine clinical decision-making, avoid futile immunosuppression in non-inflammatory phenotypes, and accelerate personalized therapy in those with refractory synovitis.

## 6. Other Emerging and Practical Applications of Synovial Biopsy in Rheumatoid Arthritis

As introduced in the previous paragraph, increasing evidence shows that pain in RA does not always reflect inflammatory load. Following previous results, a study demonstrated that synovial lining fibroblasts can activate gene programs promoting sensory nerve ingrowth, driving pain even when synovitis is histologically mild, thereby uncoupling nociception from classic inflammatory markers [33]. Consistently, Giollo et al. reported that a substantial proportion of difficult-to-treat RA patients with persistent pain exhibit only minimal inflammatory infiltrate on biopsy, pointing toward neurogenic or non-inflammatory mechanisms underlying symptom persistence [34]. These findings indicate that synovial tissue analysis can pave the way for distinguishing persistent inflammatory refractory RA (PIRRA) and non-inflammatory refractory RA (NIRRA) patients. Biopsy data have also proven valuable in the early phases of disease, particularly in undifferentiated or “at-risk” synovitis. Lympho-myeloid aggregates, sublining macrophage expansion, interferon-regulated genes, and stromal activation signatures identified in early arthritis biopsies have been associated with progression to classifiable RA and with future need for DMARD therapy, extending the predictive capacity of serology and imaging [18]. Tissue-level inflammation has further been shown to precede full clinical manifestation and probably able to define the clinical spectrum of the disease. On that topic, ACPA-positive patients have different disease spectra if compared to ACPA-negative ones; interestingly, differences are also relevant at the histological level: biopsy studies consistently show that ACPA-positive RA is enriched in B cell and plasma cell infiltrates, higher CXCL13 and immunoglobulin gene expression, and lympho-myeloid pathotypes [35]. Conversely, ACPA-negative RA more frequently displays diffuse-myeloid or pauci-immune patterns with reduced lymphoid organization, as confirmed by early arthritis tissue cohorts and multi-omics profiling in biopsy-driven trials. These data demonstrated a graded correlation between ACPA titer and synovial immunopathology, showing progressively increased immune infiltration and ectopic lymphoid neogenesis from ACPA-negative to high-titer ACPA-positive RA [36]. This stratification underscores the biological divergence between seropositive and seronegative RA that cannot be captured solely by clinical parameters. Finally, synovial biopsy contributes important information regarding structural prognosis. High densities of CD68^+^ sublining macrophages have been repeatedly associated with more aggressive radiographic progression and greater erosive burden [37]. Synovial fibroblasts also participate directly in bone destruction through RANKL expression and osteoclastogenic pathways at the pannus–bone interface [38]. Pathotype analyses further show that lympho-myeloid synovitis is linked to worse structural outcomes compared with diffuse-myeloid or fibroid patterns, reinforcing the concept that tissue-level inflammation and stromal activation are key determinants of erosive damage [39]. Overall, these converging observations demonstrate that synovial biopsy offers a broader window into RA pathobiology—spanning pain mechanisms, early-disease evolution, serological endotypes, and erosive risk—thereby complementing its established diagnostic and therapeutic value. These concepts are schematized in Table 2.

## 7. Future Perspectives of Synovial Biopsy in RA

Looking ahead, synovial biopsy is poised to play a central role in the shift from empirical therapy to precision medicine in RA. Thanks to advances in sampling, molecular profiling, and computational analysis, several avenues may transform how we use biopsy data to tailor treatment decisions.

One promising direction is the incorporation of biopsy sub-studies in ongoing trials. For example, the trial PRECISion medicinE Across the Disease Continuum (NCT04482335) includes a synovial biopsy sub-study to test predictive signatures for therapy response [40]. Even more specifically focused is the ongoing Pathobiology-Driven Precision Therapy in RA (3TR Precis-The-RA) study, which aims to validate synovial pathotypes and molecular signatures as tools for treatment selection across multiple biologics [41]. Such designs, embedding tissue research within interventional trials, allow real-time correlation between baseline synovial phenotypes and longitudinal outcomes, accelerating biomarker validation. Another key development is the application of deep molecular profiling, as exemplified by the STRAP trial, where RNA-seq from synovial biopsies predicted responses to etanercept, tocilizumab, and rituximab with AUCs of ~0.75. Translation into a 524-gene nCounter panel preserved accuracy (AUC 0.82–0.87), showing feasibility for clinical deployment. Validation in independent cohorts such as R4RA further strengthens translational potential [23,24,25].

Proteomics analysis could represent another important field of investigation of RA synovial biopsies, although studies remain relatively few. A major advance comes from the integration of spatially resolved proteomics with single-cell RNA sequencing, where laser-capture microdissection (LCM) enabled compartment-specific isolation of synovial regions prior to high-resolution LC-MS/MS profiling [28]. This approach revealed distinct protein signatures in lining, sublining, and stromal domains, illustrating how spatial proteomic variation aligns with recognized synovial pathotypes, including lymphoid-enriched and myeloid-dominated microenvironments. Complementary label-free quantitative proteomics applied to whole synovial biopsies has similarly identified protein networks associated with inflammatory activation, including the upregulation of OLFM4 [42]. These unbiased LC-MS/MS workflows map differential protein abundance that corresponds to cellular composition and inflammatory burden—features that underlie established histological and molecular pathotypes. Although not a tissue-based analysis, recent multi-tiered proteomic work has demonstrated profound hyper-permeability of the RA synovium to plasma proteins, highlighting the necessity of tissue-resolved proteomic profiling to distinguish locally produced proteins from systemic influx [43]. Together, these studies show that current proteomic techniques—ranging from LCM-guided spatial LC-MS/MS to label-free quantitative workflows—can capture protein patterns that not only define synovial microenvironments but also the added functions of serum proteins in inflamed joints, highlighting the necessity to harmonize and better comprehend links between synovial pathotypes and associated proteomics alterations. Luckily, as datasets expand, machine learning and multimodal integration of histology, clinical data, imaging, and omics are expected to enhance predictive accuracy. Clinical trial design is also evolving, with adaptive “umbrella” or “platform” trials enabling therapy allocation based on real-time biopsy-derived stratification, a model inspired by oncology [44].

At the same time, challenges remain. Repeated biopsies must be safe and feasible, requiring further standardization of minimally invasive techniques. Multicenter reproducibility depends on harmonized protocols for tissue collection and processing, while attention to synovial heterogeneity and sampling variability is essential to ensure robust biomarker interpretation.

While still experimental, the integration of liquid biopsy approaches with synovial tissue analysis could help overcome the limitations of repeat tissue sampling. By analyzing cell-free RNA or DNA fragments and other molecular components released from inflamed synovium into the circulation or synovial fluid, it may become possible to capture the biological activity of the joint in a minimally invasive way. Such strategies would allow longitudinal monitoring of molecular changes during therapy, providing dynamic insights into treatment response or resistance. Importantly, liquid biopsies could also broaden access to precision approaches by offering alternatives to centers without advanced biopsy facilities, and by reducing patient burden while maintaining a link to the biology of synovial tissue.

In summary, the future of synovial biopsy in RA lies in its integration into biomarker-driven, adaptive clinical trials, powered by high-dimensional molecular profiling and computational modeling. Ongoing initiatives such as PRECISion and 3TR Precis-The-RA exemplify how biopsy-guided designs can accelerate translation to practice. If successful, this approach could transform RA care from trial-and-error prescribing to individualized strategies guided by synovial biology.

## 8. Conclusions

Synovial biopsy has transformed the approach to RA by providing direct access to the inflamed joint tissue. The development of standardized histopathological techniques to discriminate between chronic low-grade and high-grade synovitis by grading and validated scoring systems, such as the Krenn score, has ensured diagnostic accuracy in distinguishing primarily chronic synovitis (RA, PsA) that usually could be of high grade from cases of secondary chronic synovitis (degenerative/OA and post-traumatic arthritis/PtA) that are usually of low grade, with clear implications for therapeutic decision-making.

Beyond diagnosis, the recognition of distinct synovial pathotypes—lympho-myeloid, diffuse-myeloid, and fibroid—has added a predictive dimension. In R4RA, the dichotomic classification into B cell-rich versus B cell-poor synovitis did not predict the therapeutic response to either rituximab or tocilizumab. Instead, the trial showed that treatment response was better explained by molecularly defined pathotypes, with diffuse-myeloid synovia responding preferentially to tocilizumab and lympho-myeloid tissues exhibiting comparable outcomes with both agents. Subsequent trascriptomics STRAP studies support the genetic differences and response prediction of known pathotypes. These data taken together demonstrate that these classifications are not merely descriptive but can guide therapeutic strategies: diffuse-myeloid synovia are associated with better response to IL-6R blockade while fibroid synovia show resistance to current treatments.

In clinical practice, this dual role—diagnostic and predictive—positions synovial biopsy as a precision tool capable of tailoring treatment decisions and refining the management of difficult-to-treat RA.

## 9. Future Directions

The integration of synovial biopsy into clinical research has established its value as both a diagnostic and predictive tool in RA. The next challenge is translating this knowledge into routine practice to achieve the goals of Personalized Medicine. By combining histology with molecular profiling, biopsy enables precise patient stratification, moving treatment selection beyond empirical algorithms. Trials such as R4RA and STRAP have demonstrated that synovial pathotypes and transcriptomic signatures can predict therapeutic outcomes, while ongoing initiatives like PRECISion and 3TR Precis-The-RA aim to validate these biomarkers in real-world settings.

In this context, synovial biopsy represents a patient-centered precision approach that enables the identification of individuals with true inflammatory disease, facilitates prediction of the therapeutic strategy most likely to be effective, and minimizes unnecessary exposure to immunosuppressive agents. Integrating biopsy findings into clinical decision-making has the potential to transform RA management, translating disease stratification into a practical framework for biologically tailored therapy.

## Figures and Tables

**Table 1:** The table summarizes the pivotal gene signatures found in synovial specimens of R4RA and STRAP trials.

Pathotype/Cell Program	R4RA (Bulk RNA-Seq)	STRAP (Bulk + Single-Cell RNA-Seq)	Associated Treatment Response
B cell/Lymphoid	Enrichment of immunoglobulin transcripts, CXCL13, germinal center genes	CXCL9, CXCL10, CXCL13 enriched in B cell and Tfh-like clusters	Predictive of rituximab response
Myeloid/IL-6-driven	Upregulation of SOCS3, STAT3 targets, IL1B, CCL2	Myeloid clusters with IL-6-responsive genes (STAT3, CCL2, IL1B)	Predictive of tocilizumab response
Stromal/Fibroblast	Fibroblast and ECM-related programs (HOX genes, collagens, matrix remodeling)	Fibroblast subsets enriched in ECM and developmental genes (HOX, COL1A1, COL3A1)	Associated with multidrug resistance
General immune activation	Downregulation of interferon and chemokine genes (CXCL9, CXCL10, CXCL13) in responders	Confirmed in both B cell and myeloid subsets at single-cell resolution	Common pathway of therapeutic sensitivity

R4RA: A Randomised, open labelled study in anti-TNFa inadequate responders to investigate the mechanisms for Response-Re-sistance to Rituximab versus Tocilizumab in RA; RNA: Ribonucleic acid; STRAP: Stratification of biological therapies by pathobiology in biologic-naive patients with rheumatoid arthritis; CXCL: chemokine ligand 1; SOCS3: Suppressor of cytokine signaling 3; STAT3: Signal transducer and activator of transcription 3; IL1B: Interleukin-1 beta; CCL2: chemokine ligand 2; ECM: Extracellular matrix protein; HOX: Homebox genes; COL1A1: Collagen, type I, alpha 1; COL3A1: Collagen, type III, alpha 1; CXCL9: Chemokine ligand 9; CXCL10: Chemokine ligand 10; CXCL13: Chemokine ligand 13.

**Table 2:** Other emerging and practical applications of synovial biopsy in rheumatoid arthritis.

Domain	Key Findings	Clinical Relevance
Pain Mechanisms	- Pain does not always correlate with inflammatory burden. - Synovial lining fibroblasts can promote sensory nerve ingrowth. - Neurogenic pain may persist despite low-grade inflammation.	Distinguishes inflammatory vs. neurogenic pain; avoids unnecessary immunosuppression; explains persistent pain in D2T RA.
Early/Undifferentiated Synovitis	- Lympho-myeloid aggregates and sublining macrophages predict progression to RA. - Interferon-related and stromal signatures indicate risk of persistent arthritis. - Tissue inflammation can precede clinical classification.	Helps identify patients at high risk of developing definite RA; supports early intervention strategies.
ACPA-Positive vs. ACPA-Negative RA	- ACPA+ RA enriched in B cell-rich lympho-myeloid synovitis. - ACPA− RA shows diffuse-myeloid or pauci-immune patterns. - Higher ACPA titers correlate with stronger synovial immune infiltrate.	Confirms biologically distinct endotypes; explains therapeutic differences; refines serological stratification using tissue data.
Erosive Bone Damage	- High CD68^+^ sublining macrophages predict aggressive erosive progression. - Synovial fibroblasts drive osteoclastogenesis (e.g., via RANKL). - Lympho-myeloid pathotypes associated with worse structural outcomes.	Supports use of biopsy for structural prognosis; identifies high-risk erosive phenotype; complements imaging.

D2T: Difficult to treat RA; RA: Rheumatoid Arthritis; ACPA: Anti-citrullinated protein antibodies; CD68^+^: Cluster of Differentiation 68; RANKL: Receptor activator of nuclear factor kappa-Β ligand.

## Data Availability

No new data were created or analyzed in this study. Data sharing is not applicable to this article.
